# Evaluation of parenteral nutritional support in the surgical and medical wards of a referral teaching hospital

**DOI:** 10.1186/2008-2231-20-60

**Published:** 2012-10-19

**Authors:** Samaneh Bairami, Sepideh Elyasi, Hossein Khalili, Saeed Reza Jamali-Moghadam

**Affiliations:** 1 Department of Clinical Pharmacy, Faculty of Pharmacy, Tehran University of Medical Sciences, Tehran, Iran; 2 Central Hospital Pharmacy of Imam Khomeini Hospital, Tehran, Iran; 3 Department of Infectious Diseases, Imam Khomeini Hospital, Tehran, Iran

**Keywords:** Parenteral nutrition, Errors, Medical and surgical wards

## Abstract

**Background and purpose:**

Malnutrition is a common problem in patients who are hospitalized in surgical and medical wards. Surgical patients, geriatric populations and individuals with severe illness are more vulnerable to malnutrition during their hospitalization course.

The purpose of this study was evaluation of parenteral nutrition services in a referral teaching hospital, Tehran, Iran.

**Method:**

Medical records of 72 patients who received parenteral nutrition during one year period in different surgical and medical wards of Imam Khomeini hospital were reviewed retrospectively by clinical pharmacists. Criteria for initiation of parenteral nutrition, selection of appropriate formulation and monitoring parameters were assessed based on the American Society of Parenteral and Enteral Nutrition recommendations.

**Results:**

Based on the patients' anthropometric parameters and serum albumin levels, 4.2%, 75% and 20.8% of the patients were well-nourished, moderately malnourished and severely malnourished respectively at the hospital admission and before nutritional support. Adequate calorie, protein, carbohydrate and lipid supports were achieved in 21.1%, 32.4%, 23.7% and 10.5% of the patients respectively. About 91% of the patients experienced at least one complication of the nutritional support.

**Conclusion:**

In this evaluation, several errors in assessment, establishing goals, and monitoring of parenteral nutrition regimens have been detected. Approximately all of the patients did not receive to the trace elements supports goals.

## Background

Malnutrition is a common problem in hospitalized patients [1,2]. Surgical patients with malnutrition have around three times more postoperative complications and four times greater risk of death than well nourished patients with similar operations [3]. Metabolic support via parenteral nutrition (PN) has become an important intervention in a variety of medical and surgical conditions [4,5]. Although PN has improved patient outcomes, recent meta-analysis have raised questions about its safety and high rate of PN-associated complications [6]. Metabolic disturbances such as hyperglycemia, electrolyte abnormalities such as hypophosphatemia or hypokalemia, infection of PN catheters, liver dysfunction (presented as steatosis, steatohepatitis, cholestasis and cholelithiasis) and embolic events can occur following PN [5,7-10]. Re-feeding syndrome is a common and potentially dangerous metabolic complication of PN that can cause fatal cardiac arrhythmia, systolic heart failure, respiratory insufficiency, and hematologic derangements [11].

PN formulations are extremely complex admixtures and errors in PN can occur in selection of appropriate formulation, dose, administration method, labeling and compounding devices [12-15]. For prevention of these errors, appropriate evaluation of patient's metabolic needs, ordering, preparation, administration and monitoring of PN is essential. Nutritional support team is responsible for evaluation and calculation of patient's metabolic needs, selecting appropriate route of nutritional support, ordering patient's specific admixture, and patient's monitoring for efficiency and complication of nutritional support [16-18]. The purpose of this study was evaluation of PN services in the surgical and medical wards of a referral teaching hospital, Tehran, Iran.

## Methods

Medical records of 72 patients who received PN in the surgical and medical wards of Imam Khomeini Hospital were reviewed retrospectively by clinical pharmacists during one year period. The Institutional Review Board (IRB) and the Medical Ethics Committee of the hospital approved the study.

Patients' nutritional assessment prior to initiation of PN, daily PN formula, and monitoring parameters were extracted from the patients' medical charts. Criteria for initiation of PN, selection of appropriate formulation and monitoring parameters were assessed based on the American Society of Parenteral and Enteral Nutrition (ASPEN) recommendations [4]. These recommendations have been summarized in Table 1[3]. Nutritional support was considered appropriate if the patient had received 90% of the recommended goals.

**Table 1:** Recommended criteria for parenteral nutrition*

**1.**	**Assessment**
	**A**. **Nutritional history**
	Medical & surgical history
	Psychosocial history
	Socioeconomic status
	Diet history (include weight changes and food preferences and intolerances)
	Medications
	Functional capacity
	**B. Physical examination (include anthropometrics)**
	**C. Biochemical assessment & other monitoring parameters**
Body weight	
	Serum electrolytes (sodium, potassium, calcium, magnesium, phosphorus, chloride, bicarbonate, blood urine nitrogen (BUN), creatinine
	Glucose
	Albumin and prealbumin
	Triglycerides
	Complete blood count (CBC)
	Liver function tests (AST, ALT, alkaline phosphatase, bilirubin)
	International normalized ratio (INR), prothrombin time
**2.**	**Establish nutritional goals**
	**A. Therapeutic plan**
	1. *Energy:* total calories 20–30 kcal/kg/day
	(hospitalized patient 20–25kcal/kg/day
	moderate stress/ malnourished 25–30 kcal/kg/day
	severe stress, critically ill 30–35 kcal/kg/day)
	2. *Protein:* maintenance 0.8-1 g/kg/day
	catabolic patients 1.2-2 g/g/day
	chronic renal failure (renal replacement therapy) 1.2-1.5 g/kg/day
	acute renal failure + catabolic 1.5-1.8 g/kg/day
	3. *Fat:* 15–30% of non-protein calories intake (≤ 2.5 g/kg/day)
	4. *Carbohydrate*: 70–85% of non-protein calories intake (≤7 g/kg/day)
	**B. Fluid:** 30**–**40 ml/kg
	**C. Additives**
	1. *Electrolyte and minerals:*
	Sodium 1–2 mEq/kg/day
	Potassium 1–2 mEq/kg/day
	Calcium 10–15 mEq/day
	Magnesium 8–20 mEq/day
	Phosphorus 20–40 mmol/day
	Acetate and chloride: as needed to maintain acid–base balance
	2. *Trace elements:*
	Chromium 10–15 mcg/day
	Copper 0.3-0.5 mg/day
	Manganese 60–100 mcg/day
	Selenium 20–60 mcg/day
	Zinc 2.5-5 mg/day
	Iron: not routinely added
	3. *Vitamins:*
	Vitamin A 3300 IU/day
	Vitamin D 200 IU/day
	Vitamin E 10 IU/day
	Vitamin K 150 mcg/day
	Vitamin C (ascorbic acid) 200 mg/day
	Vitamin B_12_ (cyanocobalamin) 5 mcg/day
	Vitamin B_1_ (thiamin) 6 mg/day
	Vitamin B_2_ (riboflavin) 3.6 mg/day
	Vitamin B_3_ (niacin) 40 mg/day
	Vitamin pantothenic acid 15 mg/day
	Vitamin B_6_ (pyridoxine) 6 mg/day
	Biotin 60 mcg/day
	Folic acid 600 mcg/day
**3.**	**Monitoring**
	**A. Daily**
	Body weight
	Vital signs
	Fluid intake
	Nutritional intake
	Output
	Serum electrolytes (sodium, potassium, chloride, bicarbonate) BUN, creatinine
	Glucose
	**B. Two or three times a week**
	CBC
	Calcium, magnesium, phosphorus
	**C. Weekly**
	Albumin & prealbumin
	liver Function tests
	INR, prothrombin time
	Nitrogen balance

*These criteria are based on the American Society of Parenteral and Enteral Nutrition recent recommendations. In this guideline baseline nutritional assessment of patients based on the anthropometric parameters, nutritional history, physical examinations, laboratory and biochemical parameters and pre-existing diseases were recommended. Nutritional requirements including calorie, carbohydrate, protein, lipid, vitamins and trace elements were considered with respect to severity of patients' illnesses and clinical status. Monitoring parameters during nutritional support course have been shown in the end of the table.

Statistical Package for the Social Sciences (SPSS) version 11.5 (SPSS Inc., USA) was used for descriptive statistical analysis. Categorical variables were expressed as percentage. Continuous data were reported as mean ± standard deviation (SD). Pair sample t-test was used for comparison of patients' laboratory parameters before and after initiation of PN. Pearson chi-square was used for evaluation of correlations.

## Results

Seventy- two patients (43 males and 29 females) received PN during one year period in the surgical and medical wards of Imam Khomeini hospital. The patients were under nutritional support only from PN (total parenteral nutrition; TPN) route. Average age of the patients was 57.3±16.6 years old. Gastrointestinal surgeries (52.8%), tumor resection surgeries (27.1%) were the most common reasons of the patients' hospital admission followed by cardiac (8.6%) and renal (7.1%) disorders. Most of the patients were admitted in the surgical wards (73.6%). The most common baseline diseases of the patients have been shown in Table 2. Forty-eight (66.7%) and 24 (33.3%) of the patients received their PN supports through a central venous catheter and peripheral venous catheter respectively. Average length of the patients' hospitalization and duration of PN were 21.3±11.0 and 11.7±7.8 days respectively.

**Table 2:** Baseline diseases of the patients*

**Disease**	**Frequency (%)**
Cancer	60.5
Diabetes Mellitus	26.3
Cardiovascular Diseases	18.4
Renal diseases	5.3

*In this table baseline diseases of the patient have been shown. Most patients who required nutrition support, suffered from cancer (especially gastrointestinal one).

Based on the anthropometric parameters (weight, high, age, sex and body mass index) and patients' serum albumin levels, 4.2%, 75% and 20.8% of the patients were well-nourished, moderately malnourished and severely malnourished respectively at hospital admission and before PN initiation.

Average basal total energy expenditure, carbohydrate, protein and lipid requirements and intakes of the patients have been shown in Table 3. Sufficient energy, protein, carbohydrate and lipid support were met in 21.1%, 32.4%, 23.7% and 10.5% of the patients respectively. Patients' mortality rates were not significantly different between the patients with sufficient or insufficient metabolic support (p =0.7).

**Table 3:** Average basal energy expenditure and other macro-nutrients requirements of the patients*

**Parameter**	**Patients' requirement**	**Patients' intake**	**P- value**
Basal energy (kcal/day)	1844.2±345.1	1045.1±457.8	<0.001
Protein (g/day)	92.5±20.1	61.1±26.9	<0.001
Carbohydrate (g/day)	308.1±108.4	173.6±17.3	<0.001
Lipid (g/day)	513.1±748.7	496.1±76.5	0.9

*Basal energy, protein, carbohydrate, and lipid requirements of the patients were calculated based on the American Society of Parenteral and Enteral Nutrition recommendations and patients' clinical status and severity of the baseline illnesses. Amount of the patients' intake were calculated based on the administered solutions that had been recorded in the patients' medical charts.

The mean volume of fluids that had administered for the patients was 3571.2±986.1 ml which was sufficient for 77.8% of the patients. Normal saline 0.9% (37%), dextrose water 10% (34%) and half saline (29%) were the most used fluids. Average intakes and adequacy of electrolytes support in the patients were reviewed in Table 4. There were no significant differences between the patients' serum calcium (p =0.7), magnesium (p =0.6) and phosphorus (p =0.7) concentrations before and after PN (Table 5). Although the mean of patients' serum albumin levels was elevated after PN but it was insignificant (p =0.2). The patients received 43.9±37.1 ml of the human albumin (20%) for mean duration of 12.9±8.3 days. The most common albumin indications in our patients were hypovolemia (16.6%), edema (7.2%) and cirrhosis (5.8%). In 76.7% of the patients who received albumin during PN period, did not had the FDA approved indications. Mortality rate in the patients who received albumin with approved indications was significantly less than those without indication (p =0.003).

**Table 4:** Average intakes and adequacy of electrolytes support in the patients*

**Electrolyte**	**Average intake (meq)**	**% of the patients who received sufficient electrolyte support**	**% of the patients who received insufficient electrolyte support**
Sodium	50	2.6	97.37
Magnesium	27.12	55.3	44.7
Potassium	69.14	92.1	7.9
Calcium	6.52	47.4	52.6

*Average electrolytes intake of the patients were collected from the patients' medical records and the administered solutions. Adequacy of the electrolytes supports were determined based on the American Society of Parenteral and Enteral Nutrition recommendations and patients' clinical status and severity of baseline illnesses.

**Table 5:** Comparison of the patients' serum electrolytes, total protein, albumin, creatinine and urea levels before and after parenteral nutrition*

**Parameter**	**Before parenteral nutrition**	**After parenteral nutrition**	**P value**
Calcium (mg/dl)	8.3±0.9	8.3±1.1	0.7
Magnesium (mg/dl)	2.3±0.5	2.2±0.4	0.6
Phosphorus (mg/dl)	2.9±0.9	2.9±1.1	0.7
Albumin (g/dl)	3.3±0.7	3.4±0.7	0.2
Protein (g/dl)	6.3±1.1	6.4±1.2	0.5
Urea (mg/dl)	53.2±47.3	50.3±47.2	0.3
Creatinine (mg/dl)	1.4±1.1	1.4±1.2	0.9

*The patients' laboratory and biochemical parameters before and after parenteral nutrition support were collected from the medical records and were compared with paired sample t-test analysis.

All of the patients who vitamins were selected as a component of their PN regimen, received more than the recommended amounts of vitamin A, D, E and B_12_. Only 13.15% and 15.8% of them received vitamin K and B-complex in appropriate doses respectively. They also received vitamin C significantly more than their requirements (p =0.003).

We have also evaluated adequacy of PN monitoring in the patients. The results have been shown in Table 6. About 91% of the patients experienced at least one complication of the nutritional supports (Table 7). The mean blood sugar of the patients before PN was 109.3±29.4 mg/dl. Hyperglycemia occurred in 23.2% of the patients as a complication of PN. Incidence of hyponatremia, hypomagnesemia and hypokalemia were 16.6%, 10.5% and 24.5% respectively in our patients. Just 1.4% and 5.8% of the patients experienced elevation of serum hepatic transaminases (≥ 3 times upper limit normal) and bilirubin concentrations respectively. There were no significant differences between the patients' serum urea and creatinine levels before and after PN. From the included patients, twelve (16.6%) of them died. We have not found significant correlation between adequate nutritional support (calorie, protein, lipid, electrolyte and vitamins) and the patients' mortality rate.

**Table 6:** Accuracy of parenteral nutrition monitoring in the patients*

**Parameter**	**Correct monitoring (%)**	**Errors in monitoring (%)**
Daily monitoring parameters (such as temperature, patient's intake and output)	13.2	86.8
CBC monitoring (semiweekly monitoring parameter)	81.6	18.4
Calcium, magnesium & phosphorus (semiweekly monitoring parameter)	21.1	78.9
Albumin (weekly monitoring parameter)	23.7	76.3
Liver function tests (weekly monitoring parameter)	39.5	60.5

*Accuracy of parenteral nutrition monitoring were determined based on the American Society of Parenteral and Enteral Nutrition recommendations and patients' clinical status and severity of baseline illnesses.

**Table 7:** Common parenteral nutrition complications in the patients*

**Complication**	**Frequency (%)**
Hyperglycemia (%)	23.3
Hyponatremia	16.6
Hypomagnesemia	10.5
Hypokalemia	24.5
Hypophosphatemia (%)	5.3
Rise of liver function tests (%)	7.2
Catheter infection (%)	2.7

* Parenteral nutrition complications were detected based on the patients' physical examinations, clinical evaluations, and monitoring of laboratory and biochemical parameters.

## Discussion

The most common problem related to pharmaceutical preparation of PN formulations is day-to-day changes of patients' nutritional requirements following changing in their clinical and physiological conditions [16,19-21]. Improving the quality of patients' metabolic supports can be achieved with adequate nutritional assessment using a standard protocol. This is especially important in the settings such as a referral teaching hospital with high prevalence of nutritional support complexities [22].

The ASPEN standards for nutritional support, recommend that all patients who are candidate for PN should undergo nutritional assessment at baseline before initiation of metabolic support. Baseline nutritional assessments include gathering of patients' demographic data such as age, gender, weight, height, nutritional history, physical examinations including anthropometric information, and biochemical parameters (Table 1). These measurements can be used to differentiate between acute and chronic malnutrition and calculation of patients' nutritional requirements [4]. These assessment were done somewhat for most of the patients during hospitalization course and had been recorded in their medical charts.

Based on the patients' baseline nutritional assessment, a metabolic support's plan for fluids, calories, protein, fat, and carbohydrate should be designed for each patient [14]. We have used ASPEN criteria for evaluation of PN in the study. Approximately 21.1% of the patients received calorie goals, but we have not found any significant correlation between the patients' calorie intake and mortality. However sample size of the study was too small for evaluation of correlations between the parameters. Eighty percent of the patients received sufficient fluid (volume) and it seems that low calorie intake was not due to inadequate intake of volume.

Electrolytes, vitamins, minerals and other trace elements are necessary component of PN and metabolic complications can arise following inappropriate replacement of these nutrients [14,21]. From these elements, calcium was frequently replaced inappropriately for the patients. Sufficient calcium replacement is necessary as there is a significant urine calcium loss in patients who receiving PN [13,18].

Errors in replacement of vitamins were more frequent in the study and most of the patients did not receive nutritional goals with respect to these elements. Vitamin D is recommended for patients receiving PN as well as other human subjects. Metabolic complications such as hypocalcemia, hypercalciuria and negative calcium balance have been reported following long-term PN [23,24]. Most of the patients received vitamin D more than their requirement, probably due to availability of one parenteral vitamin D preparation (300,000 IU) in our hospitals which contain more than even one week requirement of a patient.

Vitamin K (as phylloquinone or menaquinones) is an essential cofactor for coagulation cascade. Moreover, there are some evidences that show roles of vitamin K in the bone and vascular health. However the only generally accepted indication in the PN regimens is prevention of bleeding. The natural phylloquinone content of commercial PN products varies extensively depending on their fat sources. High levels of this element were reported in the PN formulations with soybean oil (150–300 μg per 100 g) fat origin [25]. In year 2000, the US Food and Drug Administration (FDA) organization revised their guidelines and recommend that adult parenteral multivitamin preparations should supply 150 μg phylloquinone per day [26]. In our study most of the patients did not receive vitamin K in their PN regimen.

Baseline laboratory and biochemical parameters should be assessed prior to initiation of PN. These parameters were assessed appropriately in 60% of the patients. Except sodium and potassium, other electrolytes such as calcium and magnesium were not evaluated at baseline for 89% of the patients. Vital signs (temperature, blood pressure, respiratory rate, pulse rate), weight, and fluid input and output, serum electrolytes (sodium, potassium, chloride, bicarbonate), BUN, serum creatinine and blood glucose are recommended to be monitored daily. In more than 80% of the patients these parameters were monitored semiweekly. It may be related to limited laboratory facilities, nursing staff workload, and patient's preferences [16,27].

Complete blood count monitoring was performed semiweekly in most of the patients, but serum electrolytes levels were not monitored appropriately in more than 70% of the patients. Serum albumin levels and hepatic enzymes that are recommended to be monitored as weekly intervals were assessed more frequently in 35% of the patients. In another study conducted by Macfarlane et al. the same results were obtained [16]. These differences may somewhat related to the recommendations. For example monitoring of these parameters as daily for 2 to 3 days, then every other day for 4 days, and then every week was recommended by another guideline [28]. ASPEN guidelines recommend the weekly monitoring of these values as it is simple, less expensive and requires less-frequent blood sampling. This criterion should be changed if patient's clinical condition needs close monitoring [4,16].

In this evaluation, we have found several deficiencies in assessment, establishing goals, and monitoring of the PN regimens in the medical and surgical wards. Complete nutritional assessments were performed only for 11% of the patients and many of them did not receive nutritional goals. About 25% of the patients received sufficient calorie and protein. Due to cost and availability issues of the parenteral trace elements products, approximately all of the patients did not receive their requirements.

This study has some limitations that must be considered. First, the data cannot be generalized to other teaching hospitals because of their different settings. Second, the study was retrospective and other factors such as exact nutrition status of the patients and effects of the patients' demographic data such as socio-economic status were not evaluated. Small sample size is another limitation of the study. Also we have not followed the patients for consequence of adequate or inadequate of the nutritional support.

## Conclusion

Errors in nutritional support processes, especially in era of PN are common in the medical and surgical wards. Development of an internal PN protocol for each hospital under observation and monitoring of a nutritional support team consisted of a physician, dietitian, pharmacist and nurse can prevent these errors. Education of hospital staff about appropriate patient's nutritional assessment at hospital admission, ordering, and administration of PN solutions are also helpful.

## Competing interests

The authors have not any conflict of interest about this work and have not any financial support.

## Authors' contributions

The manuscript is results of a hospital pharmacy project. SB is main investigator of the study and was responsible for data gathering and review of the patients' charts. SE as a clinical pharmacy resident was responsible for data analysis and preparation of the manuscript draft. HK is supervisor of the study and responsible for editing and revising of the manuscript. Clinical evaluation of the patients during course of the study was done by SRJM. All authors read and approved the final manuscript.
